# Supplementary material for: Molecular mechanism of agonism and inverse agonism in ghrelin receptor
Source: Nat Commun. 2022 Jan 13;13:300. doi: 10.1038/s41467-022-27975-9 (PMC8758724; doi:10.1038/s41467-022-27975-9)
Supplement: Supplementary file 1 — Supplementary Information [file 41467_2022_27975_MOESM1_ESM.pdf]

Molecular mechanism of agonism and inverse agonism in ghrelin receptor

Supplementary information

Supplementary Figures 1-13
Supplementary Table 1-2

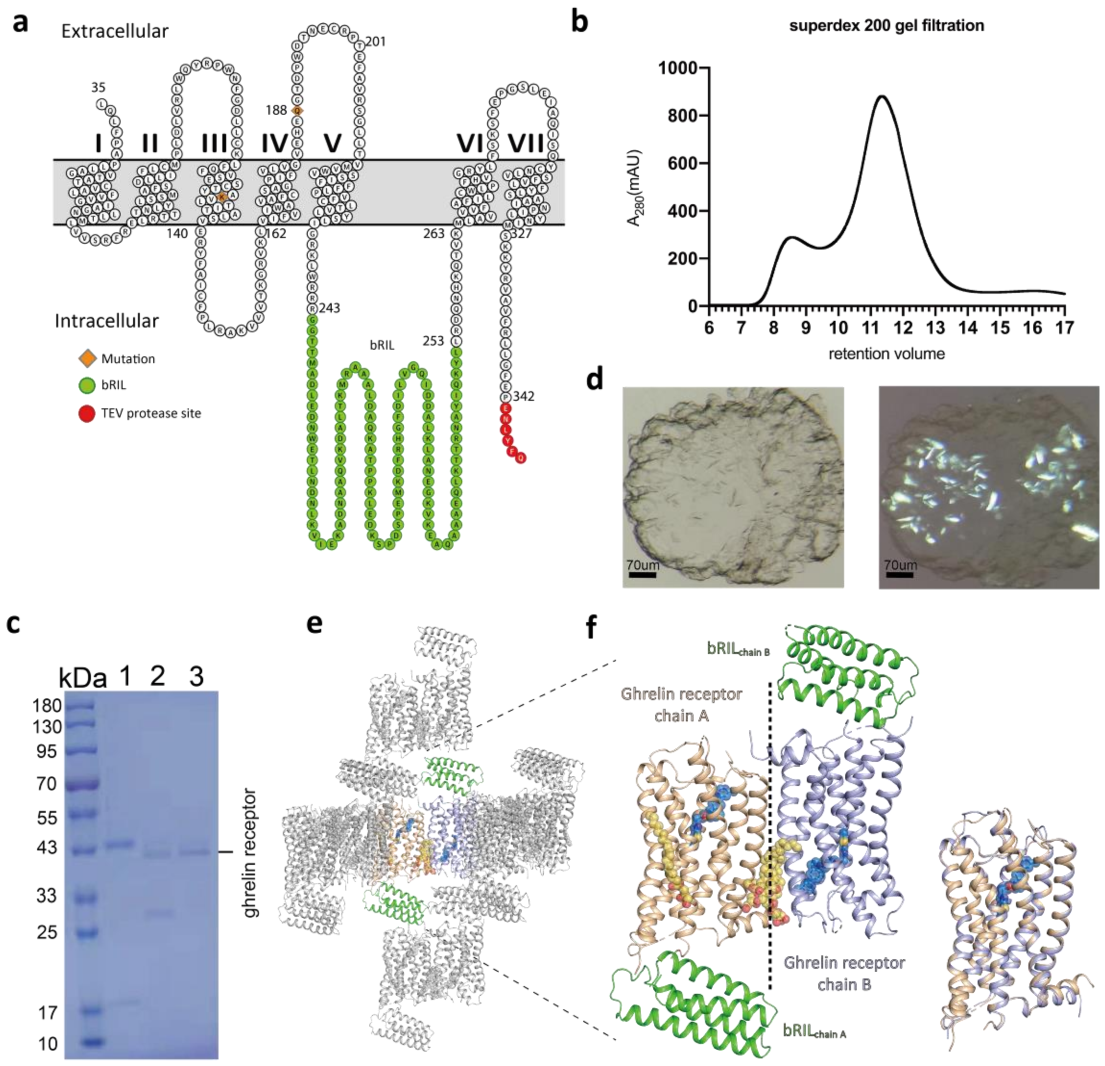

Supplementary Fig. 1: Purification and crystallization of the ghrelin receptor.

(a) Snake plot of the ghrelin receptor construct for crystallization. Residues 243-253 of ICL3 were replaced by bRIL protein. Mutations T130$^{3.39}$K and N188^{ECL2}Q is highlighted in orange color and TEV protease recognition site is shown in red color.

(b) Superdex 200 gel filtration trace for ghrelin receptor (T130$^{3.39}$K/N188^{ECL2}Q)-bRIL in the presence of inverse antagonist PF-05190457.

(c) SDS-PAGE analysis of samples in different purification steps of PF-05190457 bound ghrelin receptor (T130$^{3.39}$K/N188^{ECL2}Q)–b$_{562}$RIL purification. 3 lanes from left to right, 1: TALON IMAC chromatography purified receptor, 2: the receptor treated by TEV protease, 3: final sample after gel filtration. Marker (MW in kDa) is showing on the far left. Data is repeated over three times.

(d) White light (left panel) and polarized (right panel) microscope images of ghrelin receptor crystals in LCP droplets. Crystals can be formed over three independent experiments.

(e-f) Two molecules (termed as chain A and chain B) in asymmetric unit (e). The fusion protein bRIL is shown as cartoon with green color, and each PF-05190457 in two receptors is shown as sphere with blue color. Structural superimposition reveals the nearly identical conformation of receptor in two molecule A and molecule B (f).

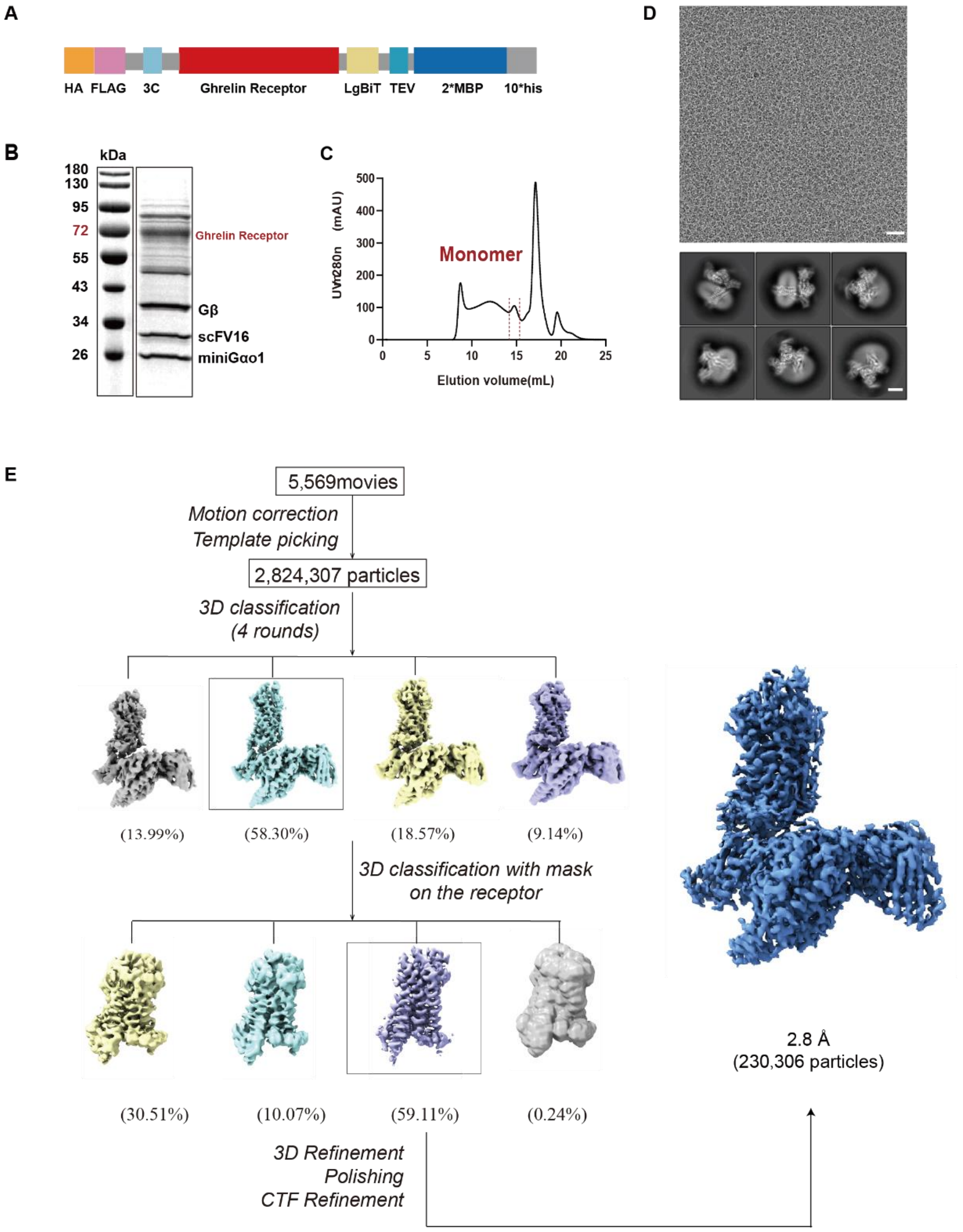

Supplementary Fig. 2: Ghrelin receptor-Go complex bound to ghrelin purification, cryo-EM data collection and cryo-EM map quality.

(a) Schematic diagram of ghrelin receptor construct designed in cryo-EM study, and NanoBiT strategy is used for ghrelin receptor-Go complex assembly.

(b) SDS-PAGE analysis of ghrelin receptor-LgBiT- miniGαo1-Gβ-HiBiT-scFv16 complexes. Samples are prepared and repeated over three times.

(c) Size-exclusion chromatography elution profiles of the purified ghrelin-ghrelin receptor-miniGαo1-Gβγ-scFv16 complex. The monomer is shown as red dotted line.

(d) Representative cryo-EM image micrographs of ghrelin-ghrelin receptor-Go complex (upper panel) and 2D class averages (lower panel), from one of the total 5569 movies.

(e) Flow chart of cryo-EM data analysis for the densities of ghrelin-ghrelin receptor-Go complex. The final resolution of the density is 2.8 Å.

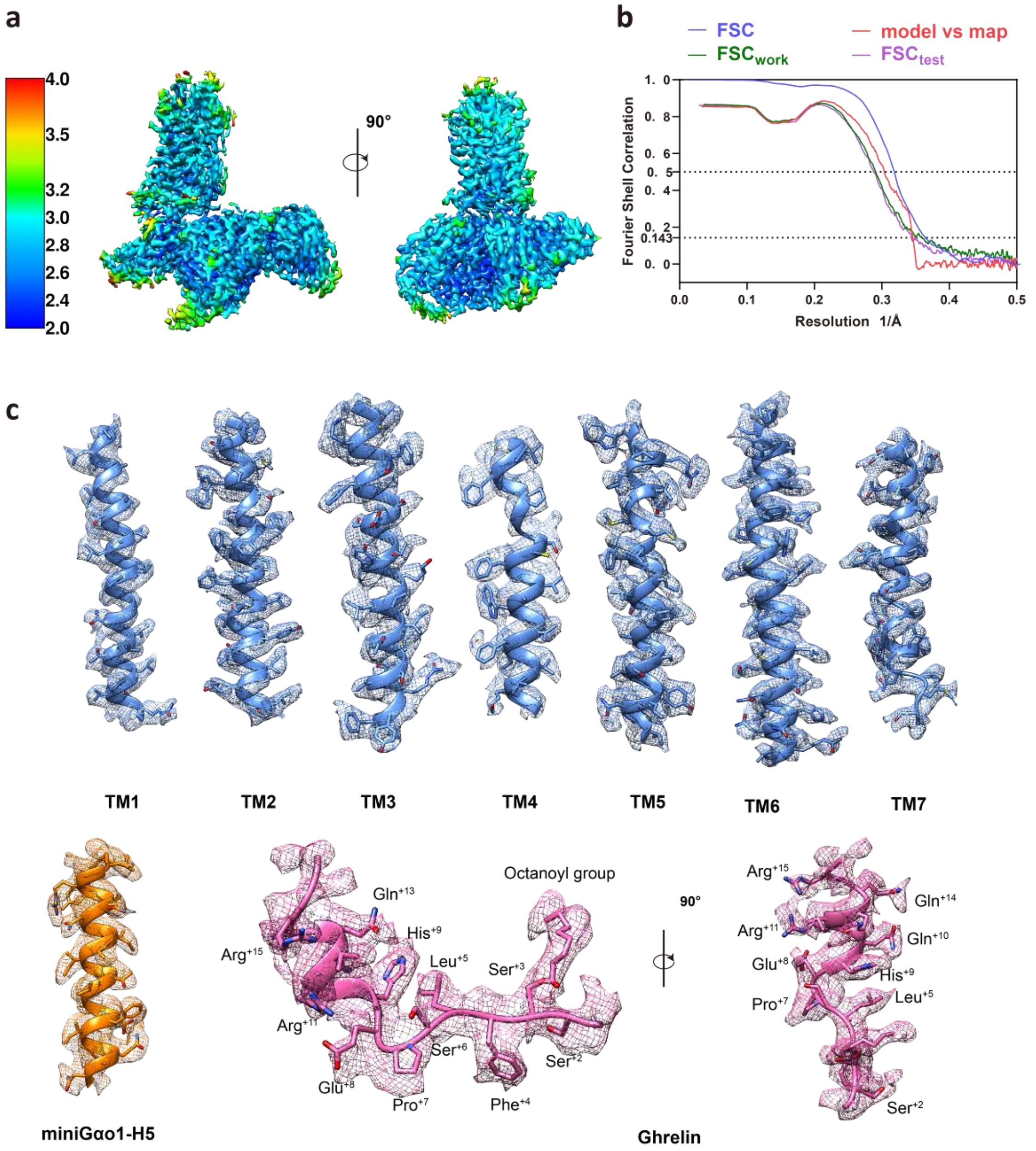

Supplementary Fig. 3: Cryo-EM map quality of Ghrelin receptor-Go complex bound to ghrelin.

(a) Cryo-EM map of ghrelin bound ghrelin receptor-Go complex colored by local resolution (A˚).

(b) The ‘gold-standard’ FSC curves， with the global resolution defined at the FSC = 0.143 is 2.8 Å for ghrelin bound ghrelin receptor-Go complex.

(c) Representative cryo-EM density maps and fitted atomic models for all seven transmembrane helixes, Gα_5-helix and the ligand ghrelin of ghrelin bound ghrelin receptor-Go complex. Ghrelin bound ghrelin receptor, blue; Gα_5-helix of miniGαo1, orange; ghrelin, pink.

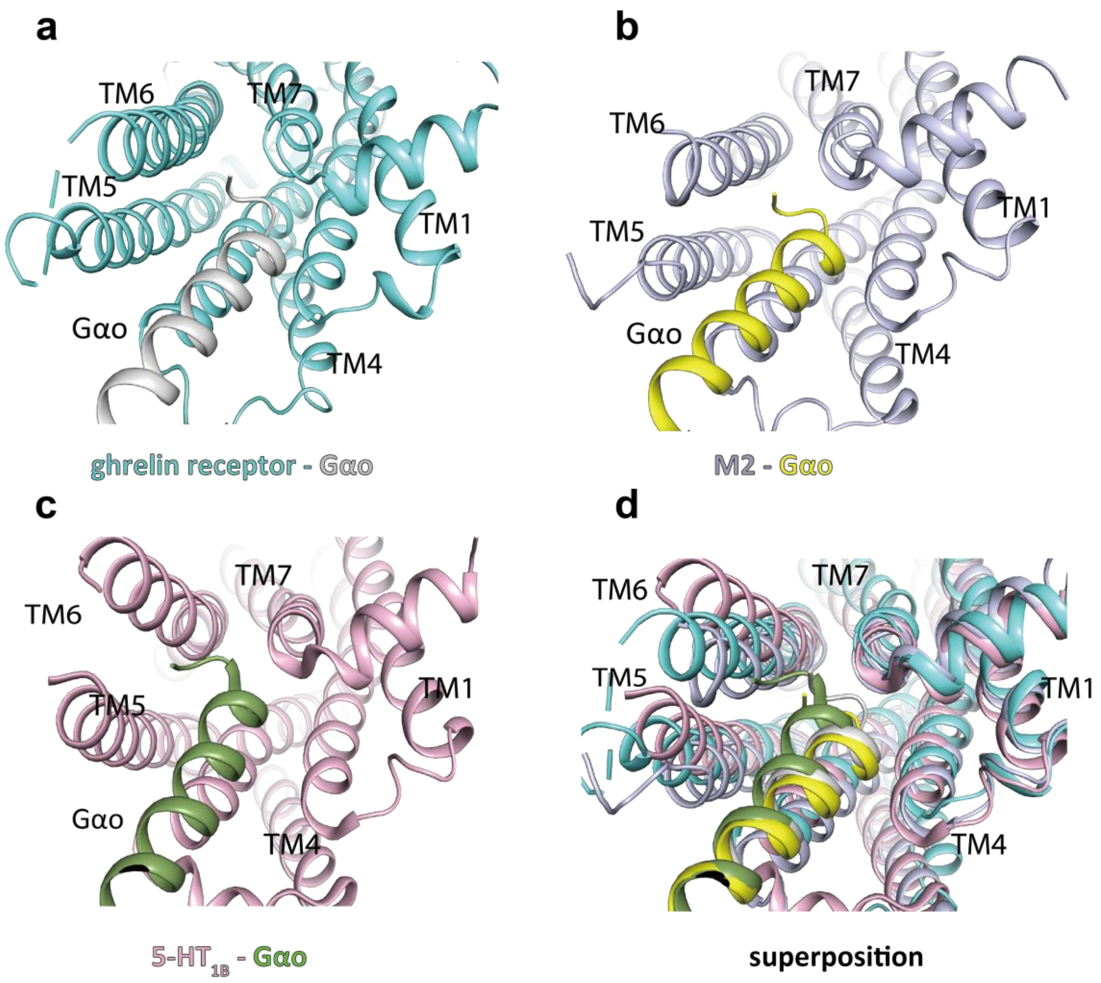

Supplementary Fig. 4: Structural comparison of ghrelin receptor-Go complex interface with other GPCRs-Go complex.

View from intracellular side of the membrane shows similar conformations compared with ghrelin receptor-Go (a), M2- Go (PDB: 6OIK) (b), 5-HT$_{1B}$-Go (PDB: 6G79) (c) and superposition of the three complex structures based on receptor alignment (d).

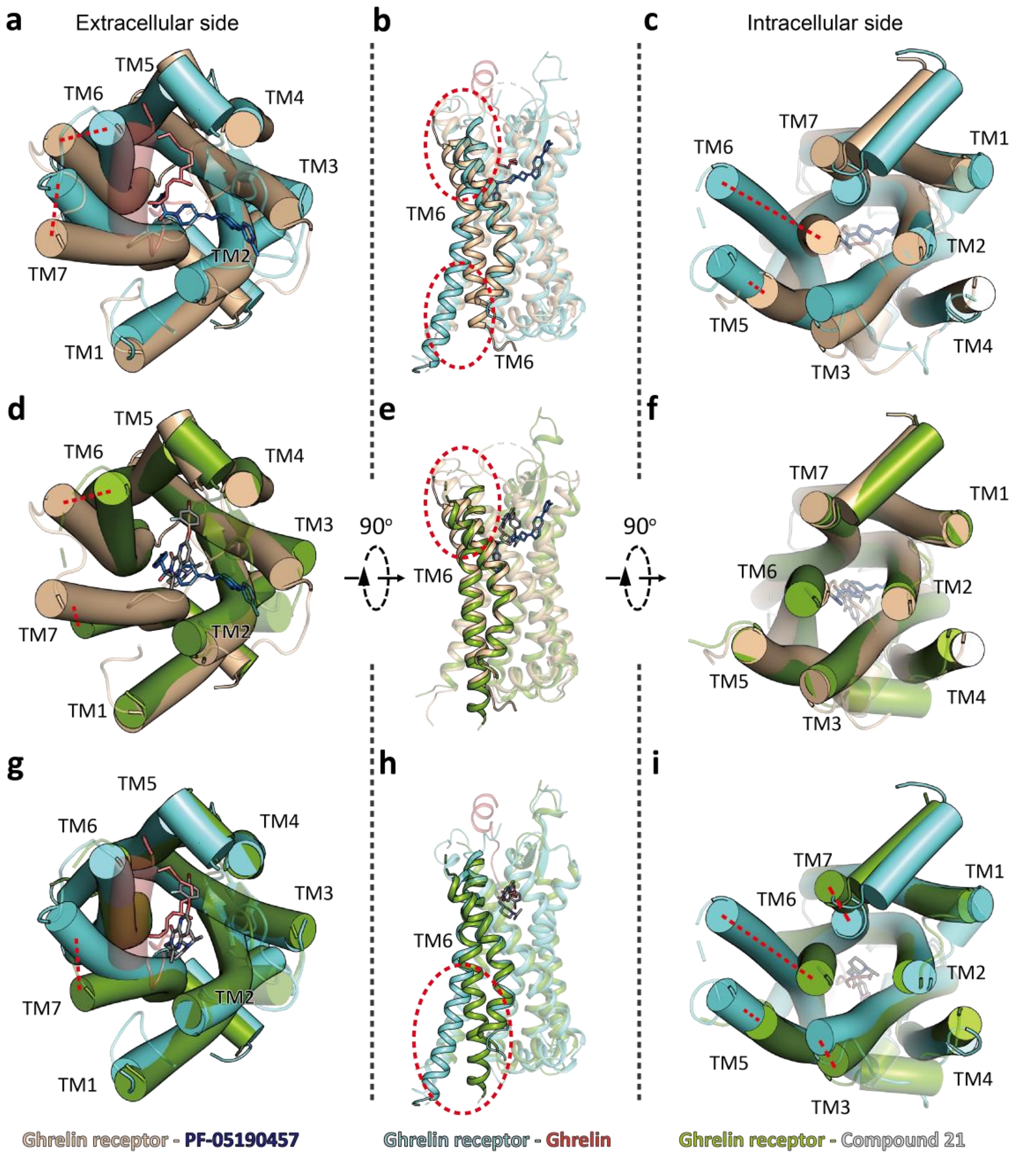

Supplementary Fig. 5: Structural comparison of ghrelin receptor with different type of ligands.

(a-c) Comparison of agonist bound ghrelin receptor (aquamarine) with inverse agonist bound receptor (wheat) reveals the notable displacement of TM5, TM6 and TM7 in extracellular side **(a)**, the plane of membrane **(b)** and intracellular side **(c).**

(d-f) Comparison of inverse agonist bound ghrelin receptor (wheat) with neutral antagonist (compound 21) bound receptor (forest) (PDB: 6KO5) reveals a significant conformational shift of TM6 and TM7 in extracellular side **(d and e)**, and shows the similar conformation in the intracellular region **(f)**, suggesting that the inverse agonist PF-05190457 stabilize the ghrelin receptor in inactive conformation.

(g-i) Comparison of agonist bound ghrelin receptor (aquamarine) with neutral antagonist (compound 21) bound receptor (forest) (PDB 6KO5) reveals that ghrelin stabilize the receptor in active conformation, as is seen from view from extracellular side **(g)**, the plane of membrane (**h)** and intracellular side **(i).**

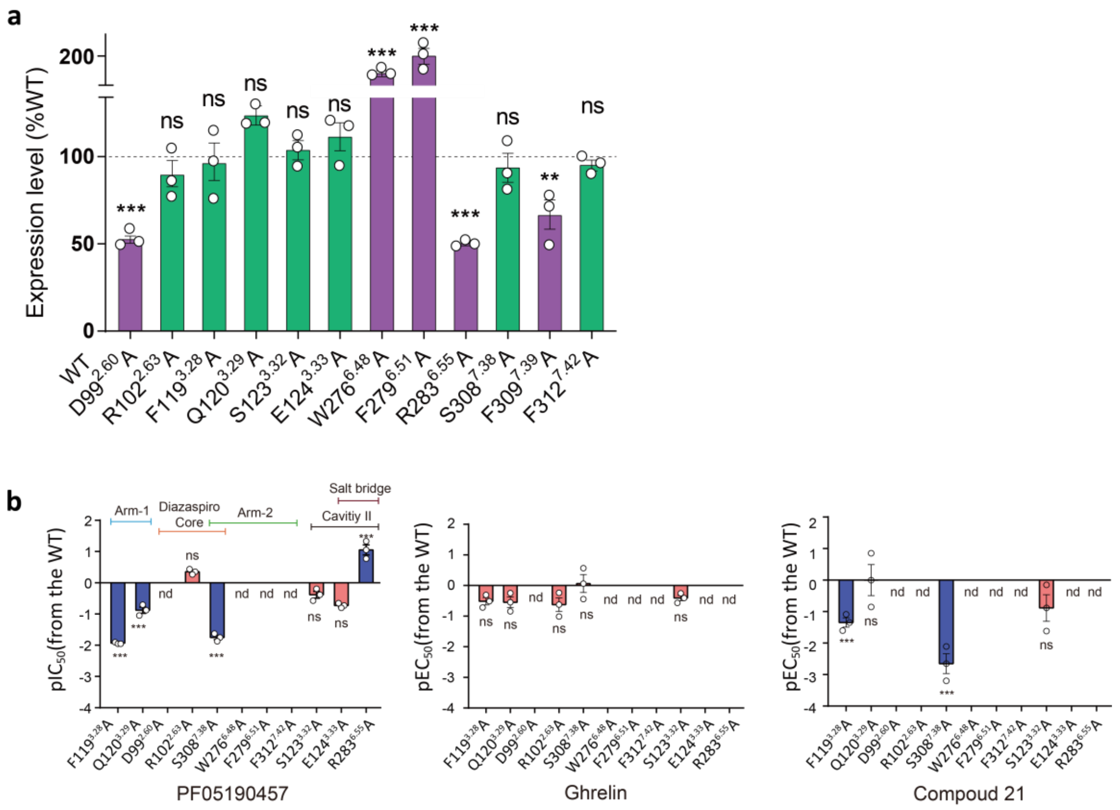

Supplementary Fig. 6: IP1 accumulation assay.

(a) Expression level of the ghrelin receptor mutants. Data represent the mean ± SEM from three independent experiments performed in triplicate. ns, not significant, $**p < 0.01$, $***p < 0.001$ (one-way analysis of variance [ANOVA] followed by Dunnett's test, compared with the response of WT, $p < 0.001$, p=0.899, p=0.999, p = 0.077, p=0.999, p=0.772, $p < 0.001$, $p < 0.001$, $p < 0.001$, p=0.996, p=0.008, p=0.999 from left to right). Data represent the mean ± SEM from n=3 biologically independent experiments performed in triplicate.

(b) IP1 accumulation measurement induced by inverse agonist PF-05190457(left), agonist ghrelin (middle) and antagonist compound 21 (right). ns, not significant; nd, not detected; $***p < 0.001$ (one-way analysis of variance [ANOVA] followed by Dunnett's test, compared with the response of WT, $p < 0.001$, $p < 0.001$, nd, p=0.05, $p < 0.001$, nd, nd, nd, p=0.055, p=0.054, $p < 0.001$, p=0.246, p=0.201, nd, p=0.124, p=0.999, nd, nd, nd, p=0.426, nd, $p < 0.001$, p>0.999, nd, nd, $p < 0.001$, nd, nd, nd, p=0.264, nd, nd from left to right). Data represent the mean ± SEM from n=3 biologically independent experiments performed in triplicate.

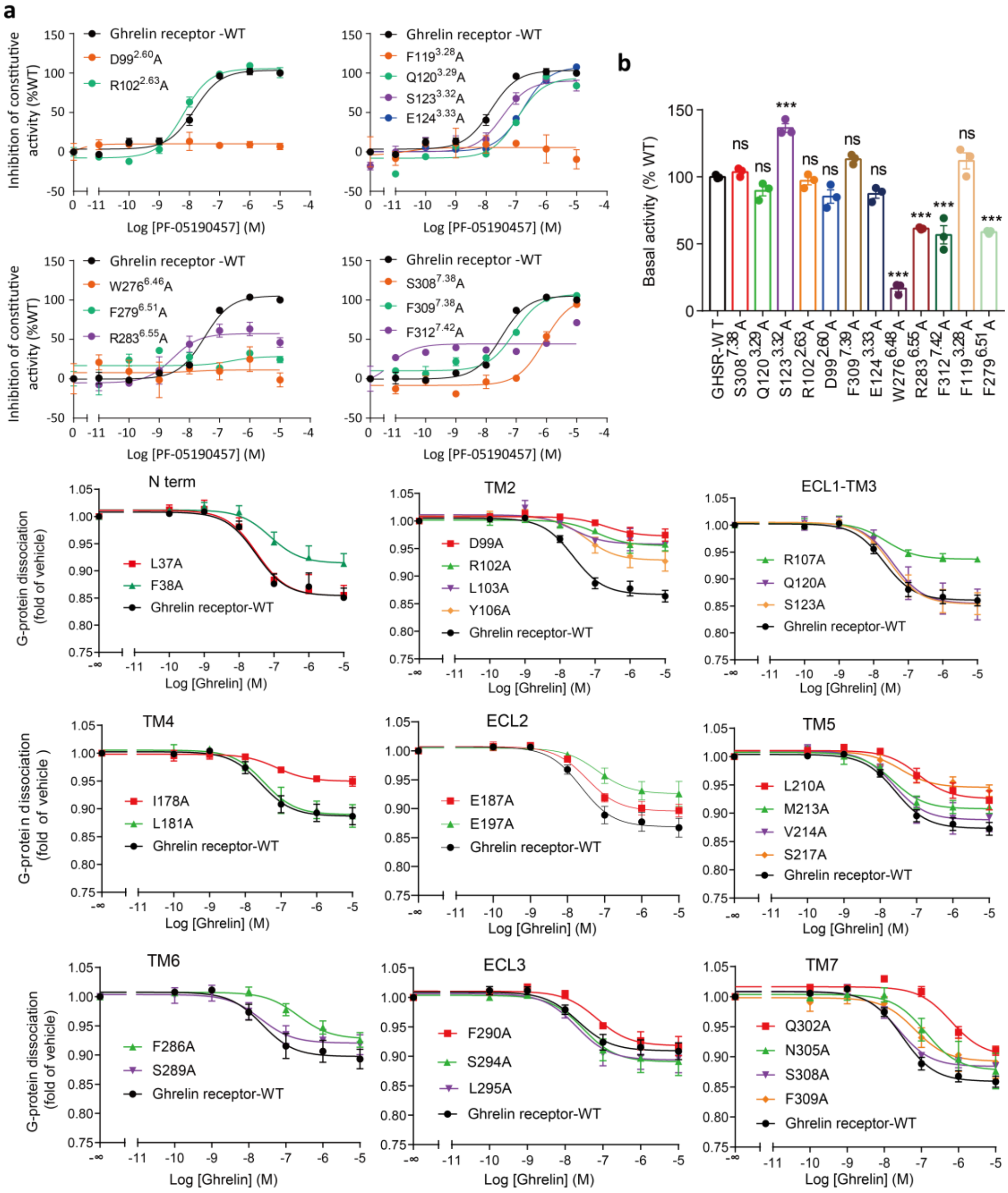

Supplementary Fig. 7: Functional assays of ghrelin receptor and mutations by the stimuli endogenous agonist ghrelin and inverse agonist PF-05190457.

(a) Representative of effects of N-terminal, TM2, TM3, TM4, TM5, TM6, TM7, ECL1, ECL2 and ECL3 residues of the ghrelin receptor on endogenous agonist ghrelin and inverse agonist PF-05190457 in IP accumulation assays and NanoBiT-Gαo/i - Gγ dissociation assay. Data represent mean ± SEM from three independent experiments performed in triplicate.

(b) The basal activity of wild-type ghrelin receptor and mutations measured by IP1 accumulation assay. Bars represents difference in calculated basal activity for mutations relative to WT of ghrelin receptor. ***$p < 0.001$ (one-way analysis of variance [ANOVA] followed by the Dunnett's test, compared with the response of WT, p=0.993, p=0.326, $p < 0.001$, p>0.999, p=0.068, p=0.055, p=0.146, $p < 0.001$, $p < 0.001$, $p < 0.001$, p=0.620, $p < 0.001$ from left to right). Data represent mean ± SEM from n=3 biologically independent experiments performed in triplicate.

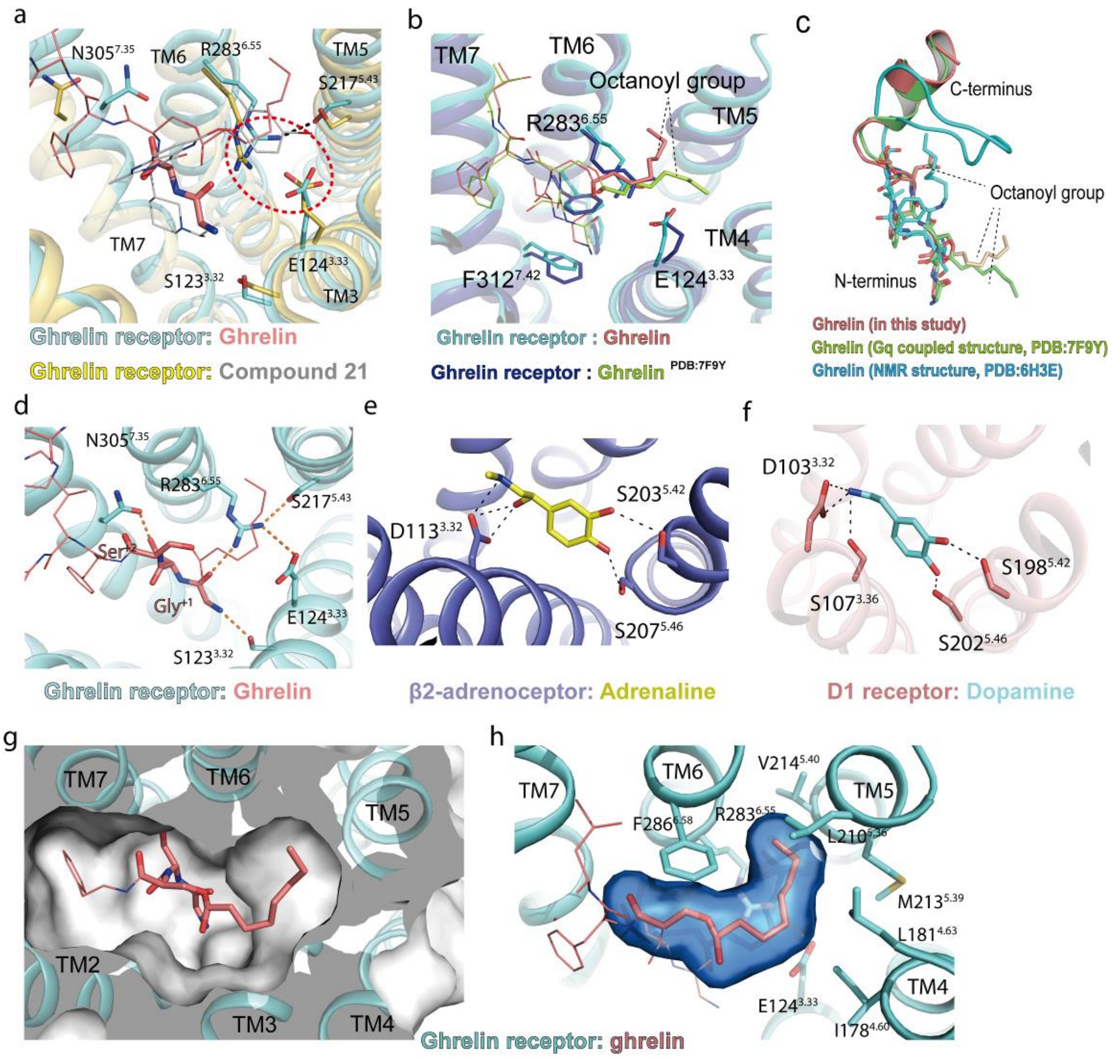

Supplementary Fig. 8: Ghrelin binding pocket of ghrelin receptor.

(a) Detailed comparison of orthosteric site in agonist bound (aquamarine) and neutral antagonist bound (yellow) (PDB: 6KO5) receptor. The obvious conformation change of $R283^{6.55}$ is observed to form additional polar interaction with $S217^{5.43}$ upon agonist binding.

(b) Comparison of the conformation of the ghrelin peptide at the N terminal, including octanoyl modification on Ser^{+3}.

(c) Superposition of the ghrelin peptide in the Gq/Go coupled receptor complex structure and solution NMR structure.

(d) The polar network that constituted by ghrelin with key residues $S123^{3.32}$, $E124^{3.33}$, $S217^{5.43}$, $R283^{6.55}$ and $N305^{7.35}$ from TM3, TM5 TM6 and TM7 respectively, appear to tether extracellular ends of TM domain and trigger activation of receptor.

(e-f) The polar network involved in activation of β_2-adrenoceptor (PDB: 4LDO) **(e)** and D1 receptor (PDB: 7CKZ) **(f)**.

(g) Cutaway surface of the cavity II in ghrelin bound structure.

(h) The obvious density map of octanoyl group in ghrelin peptide indicates the high quality. It is noteworthy that the hydrophobic cavity II accommodate to the octanoyl modification of ghrelin well.

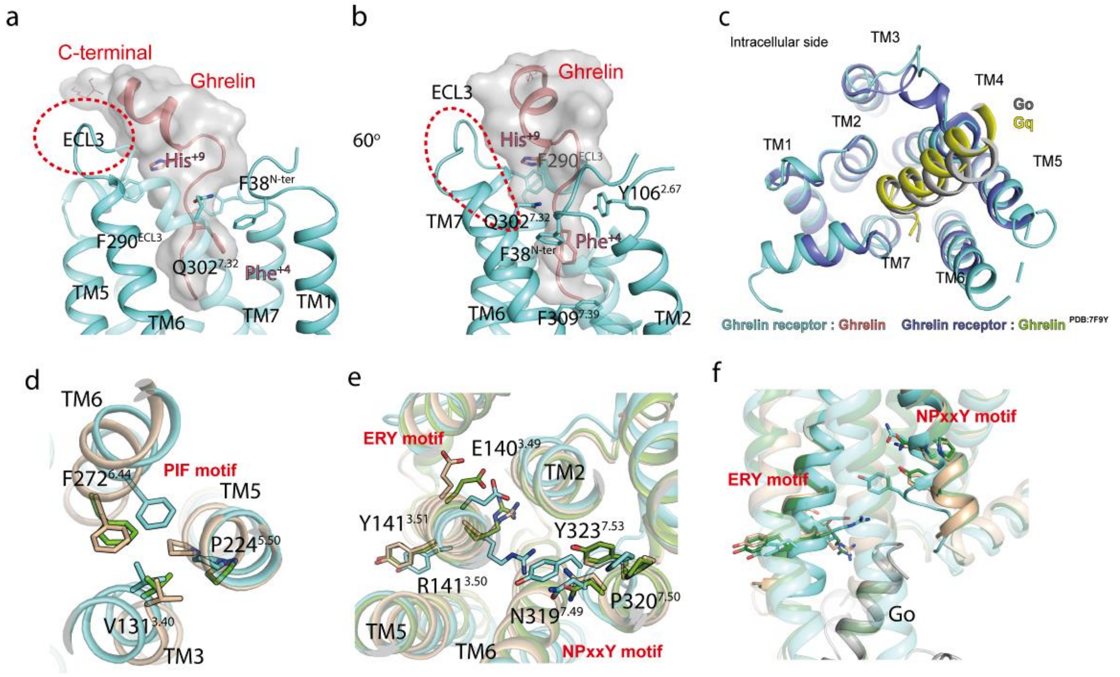

Supplementary Fig. 9: Proposed action mechanism of agonism and inverse agonism.

(a-b) The C-terminal helix of ghrelin (deep salmon) forms direct interaction with the ECL regions from ghrelin receptor (aquamarine).

(c) Structure comparison of α helix 5 of Gq or Gi protein bound with ghrelin receptor.

(d-f) Structural superposition of PF-05190457 bound (wheat), ghrelin bound (aquamarine) and neutral antagonist bound (forest) (PDB: 6KO5) receptor reveals the notable rearrangement of P-V-F motif **(d)**, E-R-Y motif and NPxxY motif **(e-f)**.

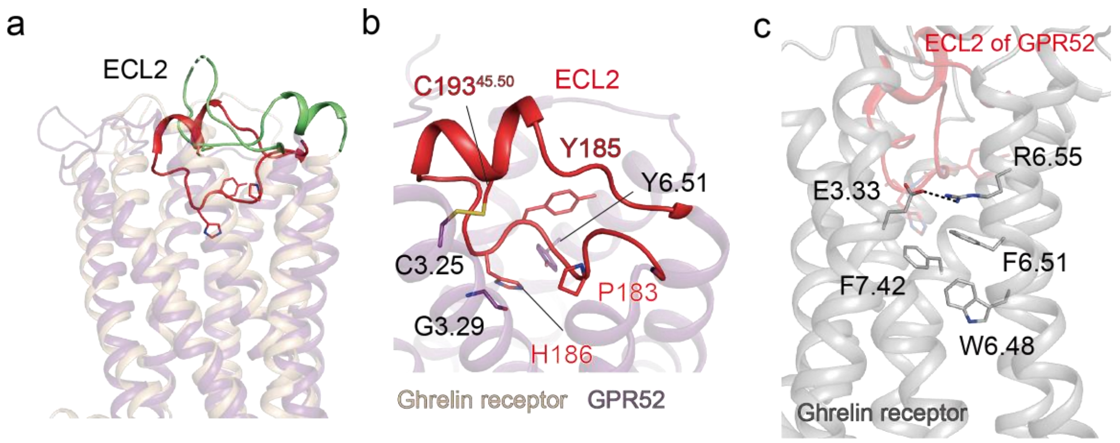

Supplementary Fig. 10: Comparison of the constitutive active mechanism of ghrelin and GPR52.

Main view (a) and Top view (b) of the active state of the GPR52 structure; (c) E-R motif and WFF cluster synergistically stable the tight contract interaction between TM6 and TM3/7.

a

GPCRs	3.33	6.48	6.51	6.55	7.42
GHSR	E	W	F	R	F
GPR39	E	W	N	R	F
NTSR2	E	W	Y	R	F
NTSR1	D	W	Y	R	F
GPR52	S	W	Y	F	A

b

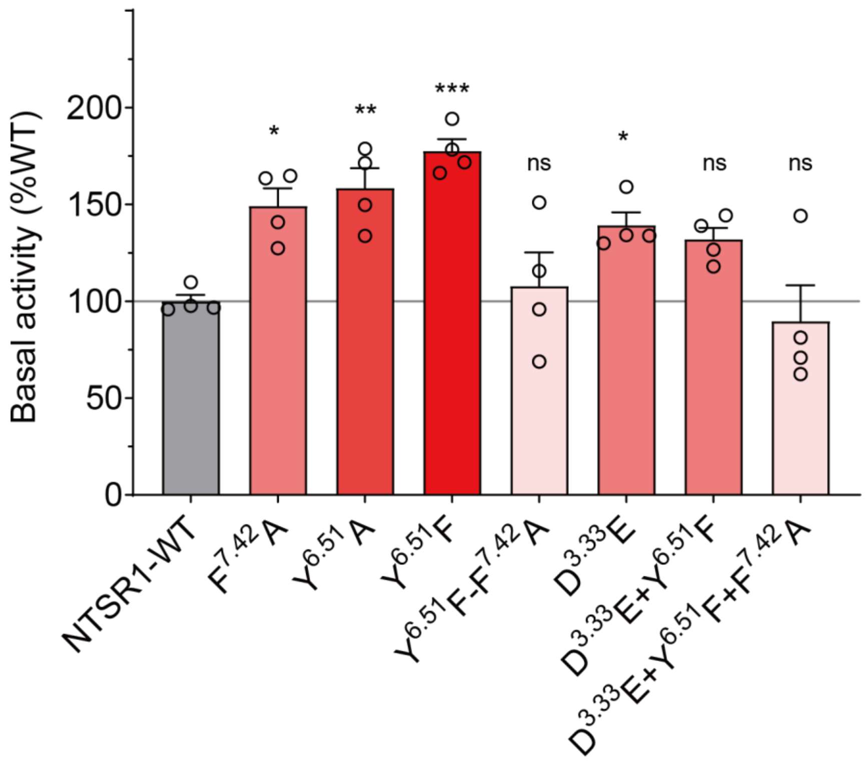

Supplementary Fig. 11: Sequence alignment of ghrelin receptor family.

(a) Alignment of the E-R motif and WFF cluster in ghrelin receptor family and GPR52.

(b) Constitutive activity of wild-type and mutant NTSR1 receptor. ns, not significant; nd, not detected; $*p < 0.05$; $**p < 0.01$; $***p < 0.001$ (one-way analysis of variance [ANOVA] followed by Dunnett's test, compared with the response of WT, $p = 0.02$, $p = 0.006$, $p < 0.001$, $p=0.994$, $p = 0.04$, $p=0.22$, $p=0.976$ from the left to right). Data represent the mean ±SEM from n=4 biologically independent experiments performed in triplicate.

a

GPCRs	3.33	6.55	6.48	6.51	7.42	5.47	6.44	6.52	7.43
GHSR	E	R	W	F	F	F	F	H	Y
DRD2	V	H	W	F	G	F	F	F	Y
ETB	K	R	W	L	A	F	F	H	S
5-HT2aR	V	N	W	F	G	F	F	F	Y
5-HT2cR	V	N	W	F	G	F	F	F	Y

E-R motif WFF cluster

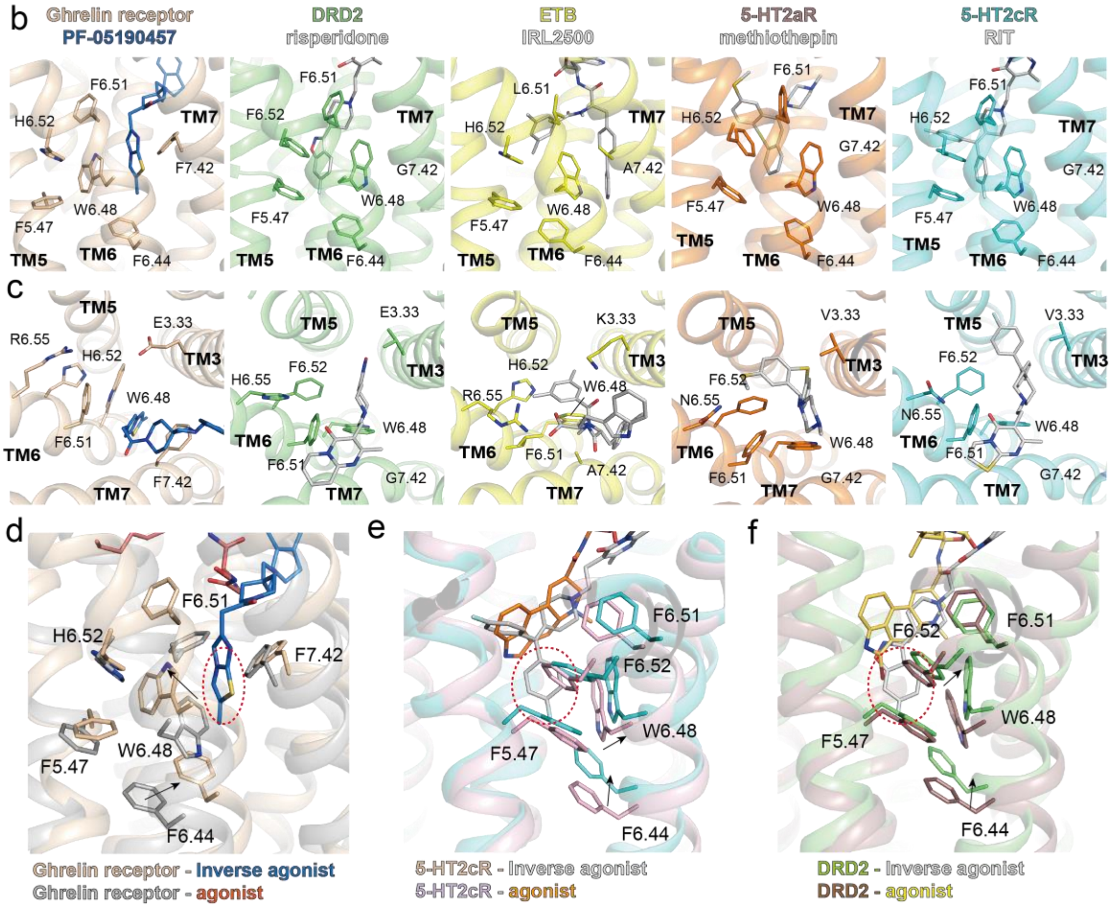

Supplementary Fig. 12: Superposition of the constitutive activity containing receptors

(a) Sequential alignment of the $E^{3.33}$-$R^{6.55}$ motif and aromatic cluster for Ghrelin receptor, DRD2, ETB and 5-HT2a/2cR.

(b) Comparison at the portion of WFF cluster for the receptors, the inverse agonists in the receptors except PF-05190457 are colored in gray.

(c)Comparison at the portion of $E^{3.33}$-$R^{6.55}$ motif for the receptors.

(d-f) Comparison between the agonist bound and inverse agonist bound ghrelin receptor structures (e), 5-HT2cR structures (f) and DRD2 structures (g).

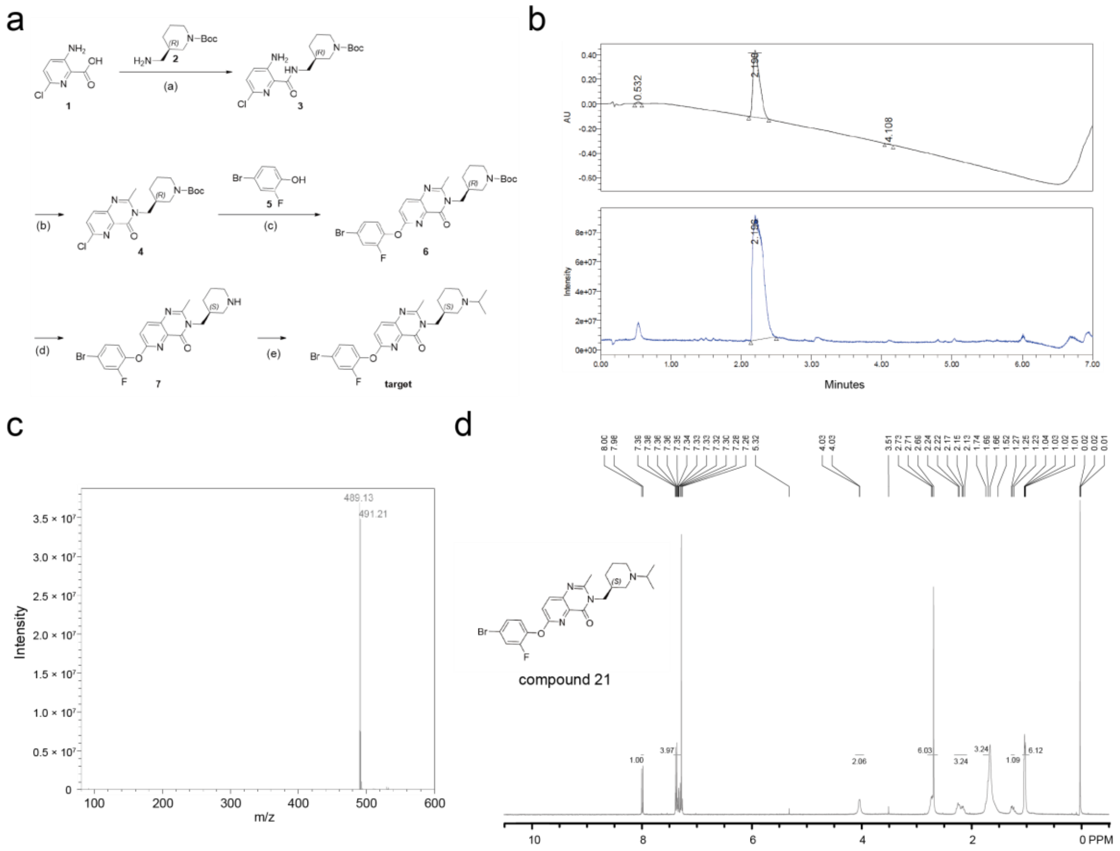

Supplementary Fig. 13: Synthesis of compound 21.

(a) Synthetic routes of compound 21. Reagents and conditions: a, 3-amino-6-chloropyridine-2-carboxylicacid, tert-butyl (R)-3-(aminomethyl) piperidine-1-carboxylate, BOP, TEA, DCM, rt, 12 h; b, compound 3, Ethyl orthoacetate, HOAc, 135 ℃, 3 h; c, compound 4, 4-bromo-2-fluorophenol, Caesium carbonate, DMF, 90 ℃, 6 h; d, compound 6, EA, rt, 0.5 h; e, compound 7, CH_3CN, 2-iodopropane, K_2CO_3, 70 ℃, 6 h. (b) MS TIC spectra of compound 21. Detect channel: W2489 ChB 220nm (top, black); QDa Positive Scan (bottom, blue). (c) MS TIC QDa Positive scan of the purified compound 21 from 80.00 to 600.00 Da at the retention time of 2.196 min. (d) NMR 1H spectra (600 MHz) of the synthesized compound 21.

Supplementary Table 1: Data collection of crystallization and refinement statistics.

PDB ID	7F83 (Ghrelin receptor with PF-05190457)
**Data Collection**	
Source, wavelength	Spring-8 BL32XU, 1.0 Å
Space group	$P2_1$
Cell dimensions	
a, b, c (Å)	84.71, 58.68, 119.24
α, β, γ (˚)	90, 90.60 ,90
Resolution (Å)	50.00 (2.94)*
R_{merge} (%)	67.8 (1919)
$I/\sigma I$	10.4 (1.3)
Completeness (%)	99.9 (100.0)
Redundancy	24.5 (23.7)
$CC_{1/2}$	99.6 (50.4)
**Refinement**	
Resolution (Å)	40.00 (2.94)*
No. reflections	25289
R_{work} / R_{free} (%)	22.7 / 26.5
No. atoms	
GHSR	4580
bRIL	1445
Ligand	74
Other (Lipid and Solvent)	128
B-factors (Å^2)	
GHSR	59.71
bRIL	122.72
Ligand	46.70
Other (Lipid and Solvent)	64.59
R.m.s.deviations	
Bonds lengths (Å)	0.006
Bond angles (˚)	1.030
Ramachandran plot statistics (%)	
Favored	96.33
Allowed	3.67
Disallowed	0.00

* Values for the highest resolution shells are shown in parentheses.

Supplementary Table 2: Cryo-EM data collection, model refinement and validation statistics

	7W2Z (Ghrelin-Ghrelin Receptor–miniGαo1)
**Data collection and processing**	
Magnification	49,310
Voltage (kV)	300
Electron exposure ($e^{-}/Å^2$)	62.24
Defocus range (μm)	-0.5 ~ -2.0
Pixel size (Å)	1.014
Symmetry imposed	C1
Initial particle projections (no.)	2,824,307
Final particle projections (no.)	230,306
Map resolution (Å)	2.8
FSC threshold	0.143
Map resolution range (Å)	2.5-4.0
**Refinement**	
Initial model used	6KO5
Model resolution (Å)	3.0
FSC threshold	0.5
Map sharpening *B* factor ($Å^2$)	-86.01
Model composition	
Non-hydrogen atoms	9118
Protein residues	1159
Lipid	2
Water	0
B factors ($Å^2$)	
Protein	57.01
Lipids	42.88
R.m.s. deviations	
Bond lengths (Å)	0.009
Bond angles (°)	1.044
Validation	
MolProbity score	1.68
Clashscore	7.81
Rotamer outliers (%)	0.71
Ramachandran plot	
Favored (%)	96.23
Allowed (%)	3.68
Disallowed (%)	0.09
