# Supplementary material for: Molecular mechanism of agonism and inverse agonism in ghrelin receptor
Source: Nat Commun. 2022 Jan 13;13:300. doi: 10.1038/s41467-022-27975-9 (PMC8758724; doi:10.1038/s41467-022-27975-9)
Supplement: Supplementary file 3 — Reporting Summary [file 41467_2022_27975_MOESM3_ESM.pdf]

nature portfolio

Corresponding author(s): Zhenhua Shao, Yan zhang, Wei Yan, Yanbin Ma

Last updated by author(s): Dec 5, 2021

Reporting Summary

Nature Portfolio wishes to improve the reproducibility of the work that we publish. This form provides structure for consistency and transparency in reporting. For further information on Nature Portfolio policies, see our Editorial Policies and the Editorial Policy Checklist.

Statistics

For all statistical analyses, confirm that the following items are present in the figure legend, table legend, main text, or Methods section.

n/a	Confirmed	
☐	☒	The exact sample size (*n*) for each experimental group/condition, given as a discrete number and unit of measurement
☐	☒	A statement on whether measurements were taken from distinct samples or whether the same sample was measured repeatedly
☐	☒	The statistical test(s) used AND whether they are one- or two-sided<br>*Only common tests should be described solely by name; describe more complex techniques in the Methods section.*
☒	☐	A description of all covariates tested
☒	☐	A description of any assumptions or corrections, such as tests of normality and adjustment for multiple comparisons
☐	☒	A full description of the statistical parameters including central tendency (e.g. means) or other basic estimates (e.g. regression coefficient) AND variation (e.g. standard deviation) or associated estimates of uncertainty (e.g. confidence intervals)
☐	☒	For null hypothesis testing, the test statistic (e.g. *F*, *t*, *r*) with confidence intervals, effect sizes, degrees of freedom and *P* value noted<br>*Give P values as exact values whenever suitable.*
☒	☐	For Bayesian analysis, information on the choice of priors and Markov chain Monte Carlo settings
☒	☐	For hierarchical and complex designs, identification of the appropriate level for tests and full reporting of outcomes
☒	☐	Estimates of effect sizes (e.g. Cohen's *d*, Pearson's *r*), indicating how they were calculated

Our web collection on statistics for biologists contains articles on many of the points above.

Software and code

Policy information about availability of computer code

Data collection	X-ray diffraction data was collected at beamline 32XU at SPring-8, Hyogo, Japan. Cryo-EM data collection was performed on a Titan Krios at 300 kV accelerating voltage in the Center of Cryo-Electron Microscopy, Zhejiang University (Hangzhou, China).
Data analysis	X-ray data was indexed, integrated, and scaled using XDS. Cryo-EM micrographs were recorded using a Gatan K2 Summit detector in counting mode with a pixel size of 1.014 Å using the SerialEM software. The ghrelin receptor structures was solved and refined using the Phenix software suite, Coot0.8.9. Signaling assays were analyzed using Graphpad Prism. MotionCor2.1, Gctfv1.18, RELION-3.0-beta2, UCSF Chimera1.14, PyMOL2.5.1 package were also used in this study.

For manuscripts utilizing custom algorithms or software that are central to the research but not yet described in published literature, software must be made available to editors and reviewers. We strongly encourage code deposition in a community repository (e.g. GitHub). See the Nature Portfolio guidelines for submitting code & software for further information.

Data

Policy information about availability of data

All manuscripts must include a data availability statement. This statement should provide the following information, where applicable:

- Accession codes, unique identifiers, or web links for publicly available datasets
- A description of any restrictions on data availability
- For clinical datasets or third party data, please ensure that the statement adheres to our policy

The structural data generated in this study have been deposited in the Protein Data Bank (http://www.pdb.org/) under accession number 7F83 [https://www.rcsb.org/structure/7F83] and 7W2Z [https://www.rcsb.org/structure/7W2Z] for the PF-05190457-ghrelin receptor and ghrelin-ghrelin receptor-Go complex. All the other data generated in this study are provided in the Supplementary information and source data files. Source data are provided with this paper.

Field-specific reporting

Please select the one below that is the best fit for your research. If you are not sure, read the appropriate sections before making your selection.

☒ Life sciences ☐ Behavioural & social sciences ☐ Ecological, evolutionary & environmental sciences

For a reference copy of the document with all sections, see nature.com/documents/nr-reporting-summary-flat.pdf

Life sciences study design

All studies must disclose on these points even when the disclosure is negative.

Sample size	Sample sizes were not predetermined by statistical methods, the size of microcrystals sample in lipidic cubic phase is determined by protein stability and overlay precipitant conditions. For cryo-EM data, sample sizes were determined by availability of microscope. Cryo-EM data was collected until we were able to refine a high-resolution structure that allowed us to obtain a high-resolution reconstruction within the confines of limited microscope time.
Data exclusions	No data was excluded from the analyses.
Replication	Protein samples were purified from different purification batch and were performed for crystallization. the final dataset is from 23 crystals, demonstrating reproducibility. Our primary data are crystal structure and cryo-EM structure that was calculated according to standard procedures and does not need replicates. The biochemical experiments in this study have been repeated by greater than or equal to three independent experiments, and those finding are reliably reproduced
Randomization	Randomization is not relevant to this study, as protein, crystal samples and our experiments did not involve choosing. Our structure was calculated according to standard procedures with freely available software and does not need randomization
Blinding	Our experiments were all biochemical studies, no blinding was used or necessary during data collection or analysis. As above, Our primary data is a crystal structure and cryo-EM structure that was calculated according to standard procedures with freely available software and did not require blinding.

Reporting for specific materials, systems and methods

We require information from authors about some types of materials, experimental systems and methods used in many studies. Here, indicate whether each material, system or method listed is relevant to your study. If you are not sure if a list item applies to your research, read the appropriate section before selecting a response.

Materials & experimental systems

n/a	Involved in the study
☐	☒ Antibodies
☐	☒ Eukaryotic cell lines
☒	☐ Palaeontology and archaeology
☒	☐ Animals and other organisms
☒	☐ Human research participants
☒	☐ Clinical data
☒	☐ Dual use research of concern

Methods

n/a	Involved in the study
☒	☐ ChIP-seq
☒	☐ Flow cytometry
☒	☐ MRI-based neuroimaging

Antibodies

Antibodies used	Antibodies used: anti-FLAG M2 HRP-conjugated monoclonal antibody (Sigma-Aldrich, Catalog Number A8592, Mouse lgG1)
Validation	The Anti-FLAG M2 HRP-conjugated monoclonal antibody is well characterized and was applied according to data sheet information details.<br>https://www.sigmaaldrich.cn/CN/zh/search/a8592-5x1mg?<br>focus=products&page=1&perPage=30&sort=relevance&term=A8592-5X1MG&type=product

Eukaryotic cell lines

Policy information about cell lines

Cell line source(s)	Sf9 cells, Expression systems, Cat. 94011S<br>HEK293 cells, ATCC, CRL-1573
Authentication	The cell lines are maintained by the supplier and verified by short tandem repeat (STR) profiling method. The cell used in this

Authentication

study are all from low-passage cell lines. Their morphology and growth curve analysis are further checked using microscope and cell counter respectively by investigators in this study

Mycoplasma contamination

The cell lines used in this study were negative for mycoplasma contamination.

Commonly misidentified lines
(See ICLAC register)

No commonly misidentified cell lines were used.
